# Intracranial Meningiomas in the Elderly: Clinical, Surgical and Economic Evaluation. A Multicentric Experience

**DOI:** 10.3390/cancers12092685

**Published:** 2020-09-20

**Authors:** Delia Cannizzaro, Maria Pia Tropeano, Ismail Zaed, Mario De Robertis, Simone Olei, Marco Vindigni, Enrico Pegolo, Daniele Bagatto, Andrea Cardia, Giulio Maira, Maurizio Fornari, Miran Skrap, Tamara Ius

**Affiliations:** 1 Neurosurgery Department, Humanitas Clinical and Research Center—IRCCS, via Manzoni 56, 20089 Rozzano Mi, Italy; delia.cannizzaro@humanitas.it (D.C.); ismaiel.mohamedhamzaalizaed@st.hunimed.eu (I.Z.); mario.derobertis@humanitas.it (M.D.R.); simone.olei@humanitas.it (S.O.); andrea.cardia@humanitas.it (A.C.); gmaira.presidenza@atenaonlus.it (G.M.); maurizio.fornari@humanitas.it (M.F.); miran.skrap@humanitas.it (M.S.); 2 Neurosurgical Unit, University Hospital of Udine (ASUFC), Piazzale Santa Maria della Misericordia, 15, 33100 Udine, Italy; marco.vindigni@asuiud.sanita.fvg.it (M.V.); enrico.pegolo@asuiud.sanita.fvg.it (E.P.); tamara.ius@asuiud.sanita.fvg.it (T.I.); 3 Department of Neuroradiology, University Hospital of Udine (ASUFC), Piazzale Santa Maria della Misericordia 15, 33100 Udine, Italy; daniele.bagatto@asuiud.sanita.fvg.it

**Keywords:** elderly patient, intracranial meningioma, neurosurgery, neurological outcome

## Abstract

**Simple Summary:**

Meningiomas are the most common intracranial tumors. Given the increase in life expectancy and the widespread access and use of brain imaging, the incidence in the elderly population (≥65 years) is continuously increasing. The risk/benefit ratio of surgery in elderly patients with intracranial meningioma has not been clearly defined because of the lack of objective measurement tools. The aim of our work to understand the risk factors associated with postsurgical outcomes and how these risk factors affected postsurgical outcomes in elderly patients with intracranial meningioma.

**Abstract:**

Meningioma is one of the most common intracranial tumors. It is benign and slow growing in the majority of cases. Given the increase in life expectancy and the number of radiological tests performed, the incidence in the elderly population (≥65 years) is continuously increasing. The surgical outcomes and prognostic factors in this age group are unclear. A retrospective analysis of all the patients treated for intracranial meningiomas in two different Italian institutions was performed. The clinical, radiological, surgical and follow-up data were retrospectively reviewed. Statistical analyses were performed to identify relationships between factors and outcomes. We also carried out an economic analysis. We analyzed 321 patients with intracranial meningioma. The mean age was 72.6 years (range, 65–90), with a female predominance (F/M, 1.41). Pre-operative deficits, cognitive impairment and seizures (*p* < 0.001) were associated with a worse post-operative Karnofsky performance scale (KPS) score (<80). A high pre-operative KPS score was associated with a good clinical and neurological outcome (*p* < 0.001). Being aged between 65 and 74 years, low surgical timing and Simpson removal grades of I and II were associated with a good outcome (*p* < 0.001). The length of hospitalization was significantly related to the outcome (*p* < 0.001). The complication rate was 14.3%. At 6-month follow-up, the mortality rate was 2.5%. The average cost was higher in patients with a pre-operative KPS score lower than 80. The outcome of intracranial-meningioma resection in elderly individuals is favorable when the pre-operative KPS score is >80. Treatment should be patient-specific, and additional factors should be considered. Patients with poor pre-operative clinical conditions might benefit from a combined strategy with partial resection and radiosurgery in order to reduce surgical timing and the complication rate.

## 1. Introduction

Meningiomas are the most common intracranial tumors, with a prevalence of approximately 97.5/100,000 [1,2] This estimate is set to increase due to the widespread access to and use of brain imaging. The number of incidental diagnoses of intracranial meningiomas is growing, representing around 30% of newly diagnosed intracranial meningiomas [3]. Moreover, due to a notable cumulative risk with age in both men and women [4], the increase in average survival brings an expanding number of patients to the attention of neurosurgical clinical practice. Different aspects influence the multidisciplinary approach to elderly patients with intracranial meningiomas: first, the general conditions and comorbidities of the patients, determining the independence level; second, the cognitive state, the presence of brain atrophy, and the location and the size of the lesion; and finally, an important aspect linked to hospitalization and intensive care time that should not be overlooked. It is a matter of fact that intensive care unit (ICU) assistance requires dedicated personal and financial effort. About 25% of hospitalized patients are admitted to ICU [5]. This entails economic and ethical repercussions. In regard to the elderly patient, even more so, the therapeutic options become a matter of profound debate: the wait-and-see strategy, partial removal, total removal and radiosurgery. We performed several analyses of the clinical, surgical, radiological and economic data of patients undergoing surgical treatment for intracranial meningioma in two Italian neurosurgery centers in the last 5 years: the Humanitas Clinical and Research Hospital of Milan and the Neurosurgical Unit University Hospital of Udine (ASUFC). Focusing on all the aspects mentioned, this retrospective study aims to outline the correct treatment of the elderly patient with intracranial meningioma in consideration of the risk–benefit ratio.

## 2. Materials and Methods

In order to fulfill the purpose of the study, a double-center, retrospective, observational, case-series study was performed.

### 2.1. Demographics

We performed a retrospective, observational, double-center, case-series study based on the investigation of data from the files of patients with intracranial meningiomas who underwent surgical procedures between January 2015 and December 2019 in two different Italian neurosurgical departments (the Humanitas Clinical and Research Center in Milan and the University Hospital of Udine (ASUFC)). The electronic databases of the two centers were retrospectively searched for intracranial-meningioma patients with histological confirmation. We investigated the demographical and clinical data: age, sex, comorbidity, pre-operative clinical and neurological conditions, use of drugs (especially anti-epileptic therapy), intra- and post-operative complications, and post-operative clinical and neurological disorders. We used the Charlson [5] comorbidity scale (CCS) to classify clinical disease. Besides these, all information about lesion features and surgical aspects of the procedures were recorded. The meningiomas were characterized according to anatomic location and histological grade. Experienced neuroradiologists evaluated the tumor size as the maximal diameter on contrast-enhanced MRI in pre-operative and post-operative images. Tumor resection was defined according to the Simpson grade-classification system. We analyzed aspects of the surgical procedure: the use of intraoperative neurophysiological monitoring and timing of the surgical procedure.

### 2.2. Follow-Up

All patients underwent follow-up with periodical clinical and neurological evaluations and radiological exams—CT and/or MRI—according to the clinical symptoms of the patients. Each patient was evaluated 1 and 6 months after the surgical procedure, in order to perform a shared analysis. The functional impairment of the elderly patients was assessed with the Karnofsky performance scale (KPS).

### 2.3. Operative Costs

Economic aspects of our case series were evaluated. To obtain an overall economic assessment, we analyzed the Italian national Medicare reimbursements table for diagnoses and procedures. The analysis took into account the length of stay (LOS) in the intensive care unit (ICU) and on the ward, type of craniotomy and surgical procedure, and presence of peri-operative and post-operative complications. We calculated the total costs of care for each patient and the average cost in two different categories based on age and pre-operative KPS score. We then evaluated the cost–benefit ratio.

### 2.4. Informed Consent

Written informed consent was obtained from all patients in agreement with the Declaration of Helsinki. The study was approved by the International Review Board (IRB) of the Humanitas Research Hospital (reference no: IRB- 1459).

### 2.5. Statistical Analysis

Numbers, percentages and averages with ranges are used to describe the outcomes and data of the descriptive statistics. Fisher’s exact test was applied to assess categorical data. *P*-values were calculated with two tails. The unpaired *t*-test was used to compare two means. QuickCalcs (2018^®^ GraphPad Software, La Jolla, CA, USA.) was used for statistical analysis. A *p*-value < 0.05 was considered significant.

## 3. Results

### 3.1. Demographic and Clinical Data

The patient demographics and clinical characteristics are summarized in Table 1.

From January 2015 to December 2019, a total of 321 patients older than 65 years were identified and included in this study. The median age was 72.6 years (range 65–90), with a female predominance (F/M, 1.41). Of our population, 58.5% (*n* = 188) was female and 41.5% (*n* = 133) was male. On admission, a mean KPS score of 91.3 (range, 10–100) was recorded. The Charlson Comorbidity Index (CCI) was calculated for each patient, with a mean index of 1.13 (range, 0–7). The main comorbidities were hypertension (41.4%), chronic obstructive pulmonary disease (10.2%), atrial fibrillation (9.6%), diabetes (7.7%) and cardiomyopathy (6.2%). No specific comorbidity was associated with a worse post-operative KPS score. Of the patients, 290 (90.3%) were symptomatic, whereas the remaining 31 (9.7%) received an incidental diagnosis. In these cases, the surgical procedure was proposed due to the tumor volume increasing over time according to serial MRI scans. No significant correlation was found between the type of onset and the post-operative KPS score (*p* = 0.93).

The most-frequently presenting symptoms were hemiparesis (7.78%), gait disturbance (7.78%), visual deficits (7.47%) and language disturbance (4.67%). Cognitive impairment was present in 15.57% of patients, and 32% of the patients (*n* =103) were receiving anti-epileptic medications prior to surgery.

### 3.2. Anatomical and Surgical Data

The anatomical and surgical data are summarized in Table 2. In 155 patients (48.3%), the left side was affected; in 147 (45.8%), the right side; and in 19 patients (5.9%), the lesion was bilateral. In 144 patients (44.9%), the meningioma was localized in the frontal lobe (including convexity and anterior cranial-base lesions). The temporal lobe was affected in 12.46% (*n* = 40), followed by the parietal lobe (11.52%, *n =* 37), planum sphenoidale (7.16%, *n* = 23), sphenoidal ridge (6.23%, *n* = 20), posterior cranial fossa (10%, *n* = 32), petroclival region (3.11% *n* = 10), anterior clinoid (2.8%, *n* = 9), occipital lobe (2.5%, *n* = 8) and cerebellopontine angle (CPA) (2.18%, *n* = 7). Intraoperative neurophysiological monitoring was used in 143 cases (44.54%) according to critical location.

The median operative time was 238 min (range, 120–699). The amount of resection was defined according to the Simpson grading system. A total resection (Simpson Grade I) was achieved in 53.8% (*n* = 173) of the patients. A total of 28.97% (*n* = 93) were classified as Grade II; 65.43% (*n* = 21), as Grade III; and 10.59% (*n* = 34), as Grade IV. After a multidisciplinary meeting, in 36 cases (11.2%), a post-operative Gamma Knife radiosurgery (GKRS) procedure was applied. Histological analysis identified Grade I in 80.68% (*n* = 259), Grade II in 17.44% (*n* = 56), and Grade III in 18.69% (*n* = 6).

Recurrence was identified in 9.3% of the cases (*n* = 30). The presence of a recurrence was associated with a higher Simpson grade for removal (III to V) (*p* = 0.004). Of the total number of recurrences, a second surgery was performed in 3.43% (11 cases). In 1.24% a postoperative radiotherapy was performed (4); in 0.62% (2), with CyberKnife; and in 0.62% (2), with Gamma Knife. There was no significant difference in outcome when complementary treatments were considered.

### 3.3. Post-Operative Complications and Deficits

The most common post-operative deficits were cranial nerve deficits (7.78%), hemiparesis (7.47%) and language disturbances (4.04%). The follow-up (6 months) mean KPS score was 89.47 (range, 10–100). Overall, the surgical mortality was 2.5% (*n* = 8) at 6 months. We report 46 post-operative complications (14%). The most frequent post-operative complications were Cerebrospinal Fluid (CSF) leakage (2.8%), hydrocephalus (2.18%) and cerebral hematoma (1.87%); 18% (*n* = 59) of the patients required intensive rehabilitation.

### 3.4. Cost Analysis

The average length of hospitalization was 12.78 days. The mean cost of intracranial-meningioma management per patient was EUR 16.577 (€). The length of hospitalization represented the greatest contributor to the total cost of intracranial-lesion treatment. Hospitalization is strongly influenced by the development of post-operative complications. We performed an analysis of the average cost, dividing the population by age into two groups: patients between 65 and 74 years and patients older than 75 years. We obtained mean costs of EUR 16.569 in the first group and EUR 16.593 in the second group. There was no significant difference in total costs in relation to the age of the patients. A second economic analysis was performed, evaluating patients with pre-operative KPS scores higher and lower than 80. We found mean costs of EUR 16.306 in the first group and EUR 18.522 in the second group. A difference of EUR 2216 was obtained in relation to pre-operative KPS score, with a cost benefit of EUR 20,258.43.

### 3.5. Statistical Analysis

The results of the statistical analysis are summarized in Table 3.

According to the analysis, pre-operative deficits (*p* < 0.001), cognitive impairment (*p* < 0.001) and seizures (*p* < 0.001) were associated with a worse post-operative KPS score (<80). Pre-operative KPS scores of 90 and 100 were associated with a good clinical and neurological outcome (KPS score > 90), and this relationship was statistically significant (*p* < 0.001). A good post-operative outcome was significantly associated with age (*p* = 0.03); good post-operative results occurred more frequently in patients younger than 75 years. From the analysis of the surgical aspects, we found statistical significance in relation to the following aspects. In terms of the surgical time, the lower the duration of the surgical procedure (*p* < 0.001), the better the outcome; Simpson Grades I and II (*p* < 0.001) were significatively correlated with good outcomes. Strong associations were observed with intra-operative and post-operative complications (respective *p*-values: *p* = 0.02 and *p* < 0.001). The length of hospitalization was significantly related to the outcome (*p* < 0.001). No significant difference was found in regard to the right side side (*p* = 0.33) and the left side (*p* = 0.23) and the histological grade of the lesions (*p* = 0.5). We stratified the patients into two different groups based on their age, comparing patients aged 65 to 74 years with those older than 75 years. The results of the statistical comparison are summarized in Table 4. There were three main differences in terms of post-operative recovery. There was a statistically significant correlation between the extent of tumor resection; Simpson Grades III and IV were associated with a poor post-operative KPS score (<80) (*p* = 0.02). The use of epileptic drugs was negatively (*p* < 0.001) correlated with a good post-operative outcome in both groups. The advent of intraoperative complications was significantly correlated with a negative outcome in the first group (*p* = 0.007).

## 4. Discussion

Increasing life expectancy distresses common clinical practice. Moreover, the greater diffusion of diagnostic exams has led to an increase in incidental diagnoses. For these reasons, in daily neurosurgical practice, more and more patients over the age of 65 are being evaluated. In regard to these patients, the therapeutic decision-making process makes uses of a tight assessment of the risk–benefit ratio. A more careful evaluation of elderly patients with intracranial meningioma must be obtained, eventually adding parameters that would not normally be used in younger patients. In elderly patients, comorbidities and the level of independence for daily activities have a major impact on the clinical and neurological outcomes, at both short- and long-term post-operative follow-up [6]. Recent studies have shown that, despite the higher post-operative mortality and morbidity rate, in elderly patients with intracranial meningioma, the surgical procedure is more beneficial than the wait-and-see strategy [7,8].

To our knowledge, this is the largest study to try to establish a correct treatment strategy for elderly patients with intracranial meningioma in consideration of the risk–benefit ratio. We performed a careful retrospective analysis of medical and surgical databases. We retrieved 321 cases of intracranial meningioma occurring in patients older than 65 years. We report a female predominance according to literature data [9]. When analyzing the comorbidities using the Charlson scale, we obtained a low index (1.2). The presence of comorbidities influences functional decline, disability, dependency, poor quality of life, institutionalization, hospitalization and death, as well as clinical and neurological post-operative outcomes [10]. Despite this, we could not observe a significant statistical difference between pre-operative comorbidities and clinical outcomes, or a difference between the two age subgroups, although the CCI score is a factor predicting decreased operative success in elderly patients with glioblastoma [11]. We suggest that the low morbidity rate in our series explains the absence of a statistical correlation. In 2015, some authors suggested that in patients older than 65 treated for intracranial meningioma, pre-operative neurological deficits are associated with a higher rate of post-operative comorbidities [12]. Our analyses highlight a statistically significant correlation between the presence of pre-operative neurological deficits and clinical neurological outcomes in the overall population (*p* < 0.001). In elderly patients, progressive cognitive decline could be appreciated in the form of cognitive impairment or even full-blown dementia [13]. Cognitive impairment hinders individuals’ daily activities due to decline of memory, attention and cognitive function. In our series, 15.57% of the patients showed Mini Mental state examinations positive for cognitive impairment. We noted a close correlation (*p* < 0.001) between pre-operative cognitive impairment and a low post-operative KPS score. In recent series including young and elderly patients, pre-operative KPS scores decreased with age. This study underlines the importance of different factors such as the extent of the removal, post-operative complications, comorbidity and tumor features in the surgery of the elderly population [14]. Unlike what was reported in the pertinent literature, the average pre-operative KPS score in our series (91.3) was significantly correlated with the post-operative KPS score (*p* < 0.001), with an overall slight non-significant decrease (mean post-operative KPS, 89.47). However, we do not observe elements of significance regarding age stratification, clarifying that age does not define a worse post-operative outcome but pre-operative clinical conditions influence it.

It is well recognized that the presence of seizures in patients with intracranial meningioma is linked to the site and size of the lesion and occurrence of cerebral edema. The resection of the meningioma leads to seizure control in most cases [15]. We analyzed the effects on the post-operative outcome of using anti-epileptic medications. We found a statistically significant correlation between the use of anti-epileptic medications (single or double drugs taken only by patients who experienced pre-operative or post-operative seizures) and a worse clinical outcome (*p* < 0.001). This observation is confirmed in the sub-analysis for age. Post-operative complications occurred in 14% of the cases. The presence of post-operative complications is strongly related with a low post-operative KPS score (*p* < 0.001). Post-operative complications are responsible for prolonged bed time, affect hospitalization length and prolong the post-operative recovery process. We analyzed the correlation between operative time and post-operative outcome in elderly patients. Our mean surgical procedure time was 238 min. Increased surgical time significantly correlates with a worse outcome. In a series of patients older than 80 years, it was also identified that the operative surgical time was a critical factor for the clinical outcome [16]. We suggest that this correlation may be linked to the effects of general anesthesia, which generally lead to longer awakening times.

In our series, in 36 cases, post-operative radiosurgery was performed, after a multidisciplinary-meeting assessment. The selection criteria for adjuvant radiosurgery were the partial removal of the tumor and a residue less than 3 cm in diameter that showed a volumetric increase at the first radiological follow-up. The adjuvant role of the Gamma Knife in reducing recurrence for residual meningiomas is well established [17,18].

In our analysis, we identified 30 cases of recurrence, with a rate lower than 10%. Pertinent literature reports a rate of aggressive course in intracranial meningioma of about 20% (in younger and older patients) [19].

The main risk factors related to recurrence are the Simpson grade of resection and histological grade.

In our results, the recurrence rate was significatively and positively associated with the Simpson grade for removal (III to V) (*p* = 0.004). Second surgery was offered to selected patients with good clinical and neurological status and surgically approachable fast-growing tumors. Patients with slow growth recurrence and suboptimal clinical conditions received radiotherapy or radiosurgery.

In the modern healthcare system, cost analysis has become increasingly important. The cost-minimization study, cost-effectiveness assessment, cost–utility analysis and cost–benefit analysis represent the main essential aspects of economic analysis [20].

Medicare-admissible reimbursements were used to estimate costs. In order to define the cost of an individual patient, we used a defined cost-allocation procedure. The cost analysis focused on the differences between the costs related to individual patients within the designated diagnoses, surgical procedure, day of hospitalization and post-operative complications. Cost analysis allows the standardization of performance assessment and clinical practice. We compared the average costs in patients aged 65 to 74 years and patients older than 75 years. We did not observe average differences in costs by age group. However, the economic analysis of the average costs stratified by pre-operative KPS score showed a difference of EUR 2216. Surgical treatment for patients who, in the pre-operative period, are not autonomous in the management of themselves and of the activities in daily practice will lead to higher healthcare costs. In conclusion, the data analysis showed that the post-operative KPS value was significantly related to age, pre-operative KPS score, intraoperative complications, the length of hospitalization, surgical timing and the grade of tumor removal. Post-operative neurological worsening is critical for poor quality of life, influencing the level of independence in daily activity. Slot, in 2017 [21], analyzed pre-operative data and clinical outcomes in older and younger adult patients surgically treated for intracranial meningioma. In this study, the authors observed different outcomes in the two groups (younger and older patients) after 6–12 months of clinical follow-up. This difference was not relevant at 12–18-month clinical evaluation. The authors, during the whole follow-up period, did not observe differences in the complication rate and mortality between both age groups.

Though advanced age may be related to poor prognosis, considering the low impact on mortality from intracranial meningiomas and the current average life expectancy, the surgical treatment of these lesions might play a crucial role. In neurosurgical strategy for elderly patients with intracranial meningioma, several factors need to be addressed. In patients with good pre-operative clinical status, the goal of surgery should be total removal when safely feasible, in order to restore a good quality of life and to avoid radiosurgery and radiotherapy. On the other hand, in patients with an already-existing degree of daily-life dependence or cognitive impairment, we must evaluate the possibility of a subtotal resection, reducing surgical times and preventing new neurological deficits, eventually making use of adjuvant therapies. Elderly-patient surgery has to guarantee a good clinical status, as their post-operative recovery compared to that of young patients is significantly more complex. For these reasons, we suggest avoiding an aggressive surgical treatment in elderly compromised patients, which would be detrimental to the clinical outcome, potentially hindering access to adjuvant therapies and rehabilitation.

## 5. Study Limitations

Our results should be interpreted contemplating the limitations of the study. Despite best efforts, the study is characterized by some limitations. The most evident is the nature of the study itself since it presents the design of a retrospective study. We considered a 6-month follow-up to share all the assessments, but this is a limitation of the study. Unfortunately, we cannot provide data regarding the patients who refused surgery; therefore, there is an element of bias within the cohort, i.e., self-selection. The cost analysis did not evaluate adjuvant-therapy or second-surgery costs, focusing the analysis on the main hospitalization period. Prospective and large-sample studies must be performed to identify prognostic factors and surgical selection criteria for elderly patients with intracranial meningioma.

## 6. Conclusions

Elderly patients are increasingly of interest in daily neurosurgical practice. Careful evaluation of intracranial meningioma patients has to take into account the clinical and neurological pre-operative status as well as the cognitive state and level of independence. Surprisingly, the pre-operative KPS score is the main influencing factor for clinical outcomes and surgical-procedure costs. The patient’s age cannot be considered a contraindication for the surgical procedure. Pre-operative planning has a major impact on post-operative outcomes and quality of life. Combined surgical and adjuvant radiosurgical treatments allow the reduction of surgical timing, post-operative complications and length of hospitalization.

## Figures and Tables

**Table 1:** Demographic and clinical data.

**Patients**	**321**
**Age**	
Mean	72.6
Range	65–90
**Sex**	
Male	133 (41.5%)
Female	188 (58.5%)
**Onset**	
Symptomatic	290 (90.3%)
Incidental (volume increasing)	31 (9.7%)
**Pre-operative KPS**	
Mean ± SD	91.73 ± 11.2
**Cognitive impairment**	50 (16%)
**Pre-operative deficit**	
Hemiparesis	25 (7.8%)
Gait disturbance	25 (7.8%)
Visual deficit	24 (7.5%)
Language disturbance	23 (7.17%)
**Anti-epileptic therapy**	103 (32.1%)

**Table 2:** Anatomical and surgical data.

Side	
Right	147 (45.8%)
Left	155 (48.3%)
Bilateral	19 (5.9%)
Localization	
Frontal lobe	144 (44.9%)
Temporal lobe	40 (12.46%)
Parietal lobe	37 (11.52%)
Planum sphenoidale	23 (7.16%)
Sphenoidal ridge	20 (6.23%)
Anterior clinoid	9 (2.8%)
Posterior cranial fossa	32 (10%)
Petroclival region	10 (3.11%)
Occipital lobe	8 (2.5%)
Cerebellopontine angle	7 (2.18%)
**Intraoperative monitoring**	143 (44.54%)
**Surgical time (minutes)**	
Mean ±SD	238.8 ± 5.2
Range	120–699
**Simpson grading system**	
Grade I	173 (53.8%)
Grade II	93 (28.97%)
Grade III	21 (65.43%)
Grade IV	34 (10.59%)
Grade V	0

**Table 3:** Factors associated with good neurological outcome.

Examined Parameter	*p*-Value
Age	0.03
Sex	0.3
Pre-operative KPS score	**<0.001**
Pre-operative deficit	**<0.001**
Cognitive impairment	**<0.001**
Charlson Comorbidity Scale score	0.17
Site	0.33
Side	0.23
Surgical timing	**<0.001**
Seizures	**<0.001**
Intraoperative complications	**0.02**
Post-operative complications	**<0.001**
Simpson grade	**<0.001**
Hospitalization days	**<0.001**
Histological grade	0.5

**Table 4:** Statistical analysis results from age stratification.

Examined Parameter	65–74	Over 75
Charlson Comorbidity Scale score	0.4	0.43
Seizures	**0.03**	**<0.001**
Intraoperative complications	**0.007**	NA
Post-operative complications	**<0.001**	**<0.001**
Simpson grade	0.1	0.02
Histological grade	0.4	0.9
Hospitalization days	**<0.001**	**<0.001**
Cognitive impairment	**0.001**	**<0.001**
